# Exploring the Mechanism of Hepatotoxicity Induced by *Dictamnus dasycarpus* Based on Network Pharmacology, Molecular Docking and Experimental Pharmacology

**DOI:** 10.3390/molecules28135045

**Published:** 2023-06-28

**Authors:** Peng Gao, Kun Chang, Shuo Yuan, Yanhang Wang, Kewu Zeng, Yong Jiang, Pengfei Tu, Yingyuan Lu, Xiaoyu Guo

**Affiliations:** State Key Laboratory of Natural and Biomimetic Drugs, School of Pharmaceutical Sciences, Peking University, Beijing 100191, China

**Keywords:** *Dictamnus dasycarpus*, hepatotoxicity, network pharmacology, molecular docking, CYP1A2, oxidative stress

## Abstract

The root bark of *Dictamnus dasycarpus* Turcz is a traditional Chinese medicine, Dictamni Cortex (DC), which is mainly used in the clinical treatment of skin inflammation, eczema, rubella, rheumatism, and gynecological inflammation. Unexpectedly, there are some cases of liver injury after the administration of DC. However, the mechanism of hepatotoxicity remains ambiguous. The aim of this study was to explore the mechanism and substance bases of DC hepatotoxicity based on network pharmacology and molecular docking, verified through pharmacological experiments. Partial prototype components and metabolites in vivo of quinoline alkaloids from DC were selected as candidate compounds, whose targets were collected from databases. Network pharmacology was applied to study the potential hepatotoxic mechanism after correlating the targets of candidate compounds with the targets of hepatotoxicity. Molecular docking was simulated to uncover the molecular mechanism. Furthermore, the hepatotoxicity of the extract and its constituents from DC was evaluated in vivo and in vitro. We constructed the “potential toxic components-toxic target-toxic pathway” network. Our results showed that the targets of DC included CYP1A2 and GSR, participating in heterologous steroid metabolism, REDOX metabolism, drug metabolism, heterocyclic metabolic processes, the synthesis of steroid hormone, cytochrome P450 metabolism, chemical carcinogens and bile secretion pathways. In vitro and in vivo experiments displayed that DC could result in a decrease in GSH-Px and oxidative stress, simultaneously inhibiting the expression of CYP1A2 and inducing hepatotoxicity. These results further indicated the mechanism of hepatotoxicity induced by *Dictamnus dasycarpus*, providing a basic theory to explore and prevent hepatotoxicity in the clinical usage of *Dictamnus dasycarpus.*

## 1. Introduction

Herbal medicines have been used to treat various diseases in China for thousands of years. At present, herbal medicines are increasingly available for clinical treatment worldwide, but many herbal medicines can induce toxicity, especially hepatotoxicity. A study performed in China found that of all drug-induced liver injuries (DILI), herb-induced liver injury (HILI) accounts for one-quarter, which should be given more attention [1].

The root bark of *Dictamnus dasycarpus* Turcz is a traditional Chinese medicine, Dictamni Cortex (DC), which is widely used in the treatment of skin inflammation, eczema, rubella, rheumatism and gynecological inflammation [2]. Unexpectedly, there are some cases of liver injury after the administration of DC. Two patients from the UK developed acute hepatitis after taking herbal preparations containing DC for the treatment of chronic skin disease [3]. In China, liver injury caused by DC occurs more frequently, such as Qingdai Pill, Keyin Pill and Zhixue Capsule, all of which can lead to hepatotoxicity [4,5]. *Polygoni multiflori* and *Dictamnus dasycarpus* were the most frequent causative agents inducing liver injury identified in a Korean study [4]. The hepatotoxicity of DC has attracted wide attention, and previous studies have reported that DC extracts can induce hepatotoxicity in mice but without explicit mechanisms and targets [5,6]. Limonoids and alkaloids are considered the main characteristic constituents [7], and research on hepatotoxicity focuses on alkaloids rather than limonoids. As the most abundant alkaloid of DC, dictamnine can potentially induce hepatotoxicity after metabolic activation by CYP3A and other pathways [8,9]. However, it is partial to research one compound for complicated compositions such as DC, whose pharmacological mechanism and toxic substance basis of the extract need to be explored further. We have reported the transformation of quinoline alkaloids from DC and their metabolites in vivo [10], constructing a basis for this study.

It is a great challenge to research the pharmacological mechanism of hepatotoxicity induced by herbal medicines due to their complex and various constituents. In addition, constituents from herbal medicines will be metabolized into more complex metabolites, making it more difficult to elucidate the pharmacological mechanism of hepatotoxicity in herbal medicines. As an important branch of network pharmacology, network toxicology has developed with the development of systems biology. Network toxicology provides a new strategy to quickly discover toxic components and explore the mechanism of toxicity by predicting and analyzing the toxic components and the targets of toxicity, aiming to construct the network of ‘toxicity-toxic components-toxic targets-toxic pathways’ [11,12]. Many studies have proven that network toxicology is a valid and reasonable method to reveal the toxicity mechanism of multiple components, targets and pathways of herbal medicines [11,13,14]. There are various mechanisms of HILI, including disordered metabolism of the cytochrome P450 (CYP450) enzyme [15,16], mitochondrial damage, oxidative stress, abnormal apoptosis [17,18] and inflammation [19]. To date, few studies have reported the hepatotoxicity mechanisms of DC, and no network toxicology studies have been conducted on it.

In this study, we selected partial prototype components and metabolites of quinoline alkaloids from DC in vivo as candidate compounds. We applied network pharmacology to study the potential hepatotoxic mechanism of candidate compounds after collecting their targets and hepatotoxicity from databases. Molecular docking was simulated to uncover the molecular mechanism. Furthermore, we evaluated the hepatotoxicity of the compounds and DC extracts in vitro and in vivo. By combining multiple approaches, we systematically studied the pharmacological mechanism and toxic substance basis of hepatotoxicity induced by *Dictamnus dasycarpus*.

## 2. Results

### 2.1. Construction of “Candidate Compounds-Targets” Network

A total of 561 targets of 36 candidate compounds were retrieved and predicted using TCMSP, Pharmapper, SwisstargetPrediction and SEA databases. The “candidate compounds-targets” network was constructed, and the degree values of nodes were calculated in Cytoscape 3.7.2 (Figure S1 and Table S1). A higher value indicates a stronger correlation.

### 2.2. Construction of a Protein-Protein Interaction (PPI) Network of DC Hepatotoxicity

**Figure 1:**
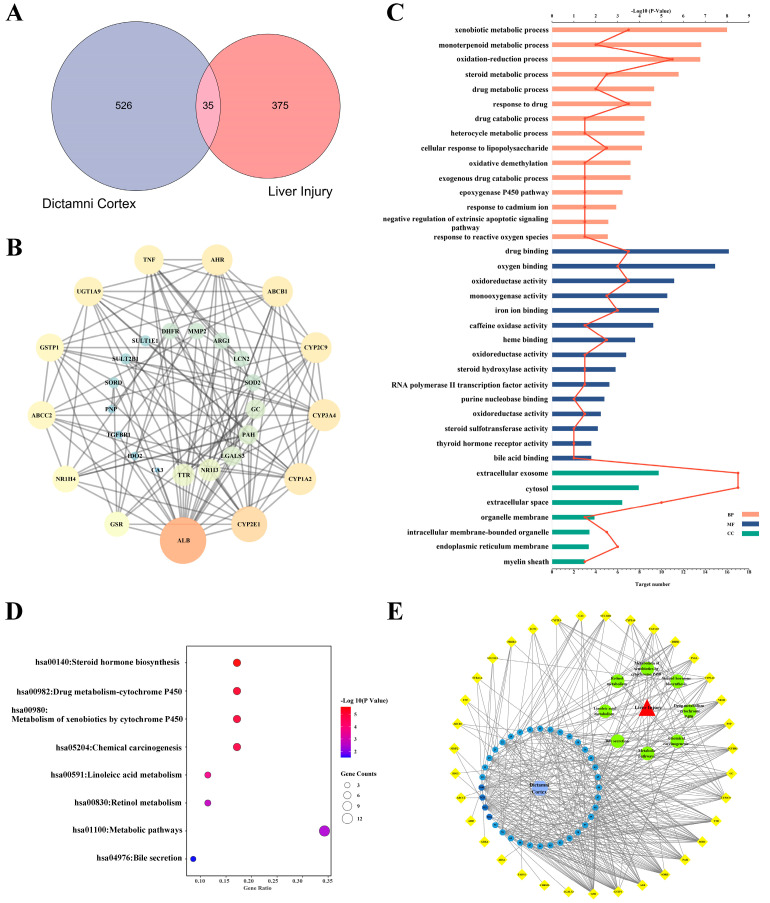
Construction of the “components-targets-pathways” network of DC hepatotoxicity. (**A**) Venn diagram of candidate compounds-targets and hepatotoxicity targets. (**B**) PPI network of 35 overlapping targets constructed based on the STRING database. (**C**) The pathways obtained from GO analysis of potential hepatotoxic targets, the histogram was value of −log_10_P and the line was number of targets. (**D**) The pathways obtained from KEGG analysis of potential hepatotoxic targets. (**E**) The network of “components-targets-pathways” of DC hepatotoxicity.

A total of 35 overlapping targets were included after comparing candidate compounds-targets and hepatotoxicity targets (Figure 1A), the detailed information of which is listed in Table S2. A PPI network was constructed after 35 targets were uploaded to the STRING database (Figure 1B). The degree values of the nodes were calculated in Cytoscape 3.7.2, and the top 10 targets, ALB, CYP2E1, CYP1A2, CYP3A4, CYP2C9, ABCB1, AHR, TNF, UGT1A9 and GSTP1, were potential key targets that played a significant role in DC hepatotoxicity.

### 2.3. Analysis of GO Pathway and KEGG Pathway

GO analysis of the 35 potential hepatotoxic targets of DC was carried out using the David database. With *p* < 0.05 as the screening condition, a total of 66 pathways were obtained, including 42 biological processes, 17 molecular functions and 7 cellular components (Figure 1C, Table S3). Among them, DC mainly participates in heterogenous metabolism, redox, drug binding, oxygen binding and so on.

KEGG analysis of the 35 potential hepatotoxic targets of DC was carried out using the David database. With *p* < 0.05 as the screening condition, eight pathways related to DC hepatotoxicity were obtained, involving steroid hormone biosynthesis, CYP450 metabolism, chemical carcinogenesis, linoleic acid metabolism, retinol metabolism and bile secretion signaling pathways (Figure 1D, Table S4).

### 2.4. The Network of “Components-Targets-Pathways” of DC Hepatotoxicity

The pharmacological network of “components-targets-pathways” was depicted using Cytoscape 3.7.2 software and is shown in Figure 1E. In the network, GSR, GSTP1, SOD, CYP, and so on were correlated with metabolites from DC.

GSR and SOD are important enzymes in the oxidative stress process. TGFBR1 is a key receptor in apoptosis, and CYP activity can affect the metabolism of drugs and biochemistry, which can induce hepatotoxicity. The concentrations of ALB, SORD and GC all indicate the degree of hepatotoxicity, and GSTP1 plays an important role in detoxification by catalyzing the conjugation of many hydrophobic and electrophilic compounds with reduced glutathione.

### 2.5. The Docking Simulation of Protein and Constituent

The key targets ALB, CYP3A4, TNF, CYP1A2 and GSR discovered through network toxicology, as well as FXR, PXR, CDK2, IDH2 and P53, which were highly correlated with hepatotoxicity in corresponding pathways, were docked with the metabolites from DC. The crystal structure of each target protein and the docking scores with the optimal ligand are displayed in Table S5. The docking scores of the candidate compounds and their target proteins are shown in Table 1.

**Figure 2:**
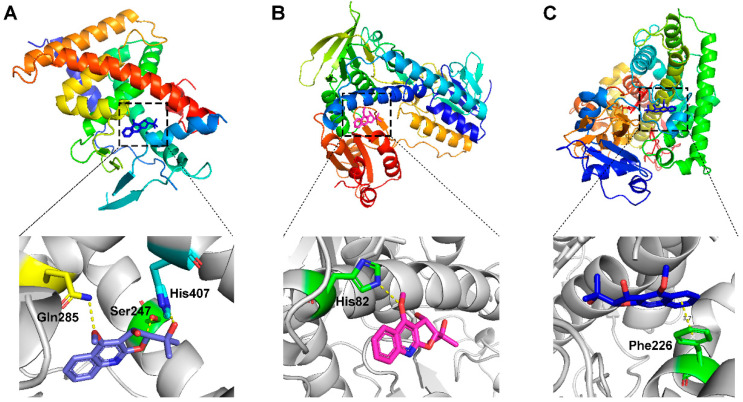
The molecular docking of compound **9** with PXR (**A**), GSR (**B**) and CYP1A2 (**C**).

The results indicated that the docking score of compound **9** with PXR was higher than that of the ligand. The docking scores of compounds **1**, **4**, **7**, **9**, **10**, **11**, **13**, **15**, **19**, **34** and **35** with GSR were higher than those of the ligand. The docking scores of compounds **4**, **7**, **9**, **10**, **13** and **19** with CYP1A2 were higher than those of the ligand. Compounds **4**, **7**, **9**, **11**, **13**, **15** and **19** were dihydrofurans that could bind to the target protein via hydrogen bonding and π–π interactions. When a hydroxyl group was present at position C-3, the number of hydrogen bonds increased, strengthening the binding to the target protein. In addition, two major non-quinoline alkaloids from DC, obacunone and fraxinellone for the negative control, were selected for molecular docking. The results showed that their docking scores with targets were lower than those with ligands, which indirectly proved the reliability of the results. As shown in Figure 2, there are three-dimensional diagrams of the molecular docking of compound **9** and key targets PXR, GSR and CYP1A2.

### 2.6. Compounds from DC-Induced Cytotoxicity of HepG2 Cells

**Figure 3:**
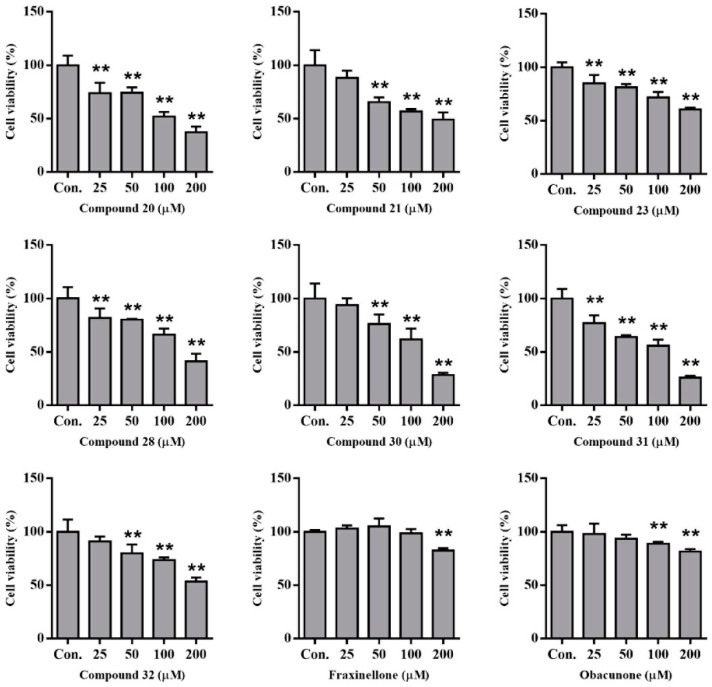
The hepatotoxicity analysis of compounds in HepG2 cells by MTT. (** *p* < 0.01, compared with control); Con.: normal control; cell viability of 1% DMSO was 90% and Adriamycin (2 µM) was 50%.

We previously isolated abundant alkaloids from Dictamni Cortex [7]. Herein, we treated HepG2 cells with several representative compounds to evaluate their hepatotoxicity. After incubation for 48 h, the cell viability of HepG2 cells was calculated using MTT analysis. The results indicated that these alkaloids induced cytotoxicity more strongly than fraxinellone and obacunone (Figure 3). Considering that the docking scores of alkaloids were higher than those of fraxinellone and obacunone, there was consistency between hepatotoxicity and docking.

### 2.7. DC Induced the Increase in Relative Liver Weight

**Figure 4:**
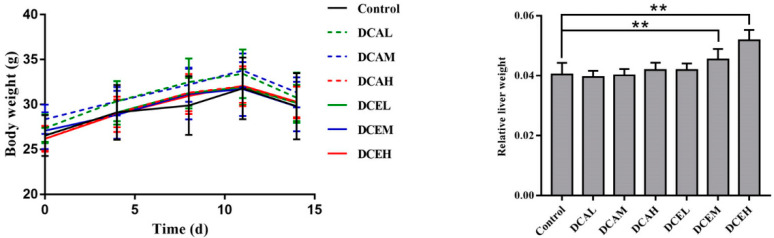
Changes in body weight during treatment and the relative liver weight of male mice. (Body weight on the last day was measured after fasting for 12 h. The relative liver weight was the liver weight divided by the body weight at sacrifice. ** *p* < 0.01, compared with control).

To estimate the hepatotoxicity of DC in vivo, mice were treated with DC for two weeks. The body weight change during treatment and relative liver weight at sacrifice were recorded. As displayed in Figure 4, there were no significant differences in body weights between the drug-treated groups and the control group. However, compared with the control group, the relative liver weights of the DCEM and DCEH groups (470 mg/kg/d and 940 mg/kg/d, respectively) significantly increased (*p* < 0.01). The results of female mice are shown in Figure S2.

### 2.8. H&E Staining of Liver Tissues Revealed DC-Induced Injury of Hepatocytes

**Figure 5:**
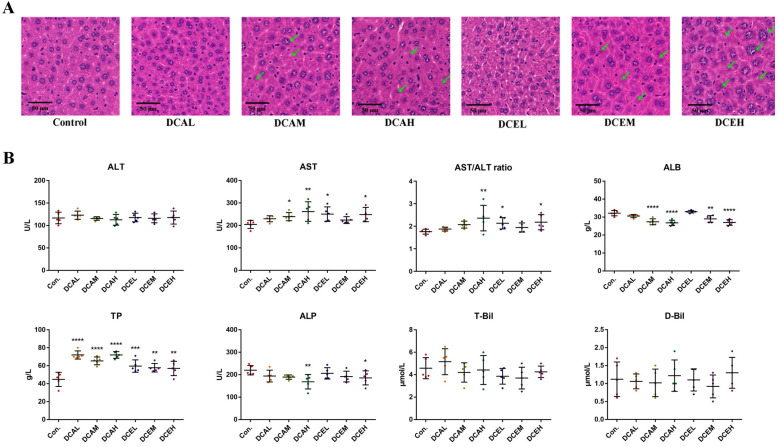
The H&E staining (**A**) and serum biochemical indexes (**B**) of male mice, green arrows indicate damaged cells. (* *p* < 0.05, ** *p* < 0.01, *** *p* < 0.001, and **** *p* < 0.0001, compared with control).

The results of H&E staining indicated that DC can lead to hepatocyte injury. Compared with the control group, the administration of DC induced nuclear swelling in the liver pathological sections, whereas partial nuclear dissolution and chromatin deepening existed in the middle and high groups (Figure 5A). No apparent sex differences were observed in our results.

### 2.9. DC Induced Oxidative Stress, Increased Serum AST and TP, and Decreased ALP and ALB

The results in Figure 5B indicate the changes in serum biochemical indexes related to liver injury after the administration of DC. Compared with the control group, the high-dose groups (DCEH and DCAH) exhibited significantly increased AST activity (*p* < 0.05) and decreased ALP activity (*p* < 0.05). All doses significantly increased the content of serum TP (*p* < 0.01). The middle- and high-dose groups (DCAM, DCAH, DCEM, and DCEH) had significantly decreased serum ALB (*p* < 0.01). No dose had a significant effect on the content of D-Bil and T-Bil. The results of females are shown in Figure S3B. These data illustrate the adverse effects of DC on the liver.

**Figure 6:**
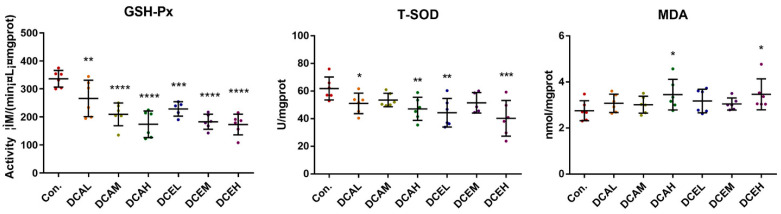
DC induced oxidative stress of liver tissue. (* *p* < 0.05, ** *p* < 0.01, *** *p* < 0.001, and **** *p* < 0.0001, compared with control).

Network pharmacology indicated that DC-induced hepatotoxicity was related to oxidative stress, so the associated oxidative stress indicators in various groups were examined. GSH-Px and T-SOD are key antioxidant enzymes, and MDA is a product of lipid peroxidation. When oxidative stress occurs, the activities of GSH-Px and T-SOD may be reduced, and the levels of MDA will increase. The levels of MDA in the liver tissues of DCAH and DCEH were also significantly increased (*p* < 0.05), whereas the activities of T-SOD and GSH-Px were remarkably decreased (*p* < 0.01) compared to those in the normal group (Figure 6).

### 2.10. DC Downregulated the Expression of CYP1A2

**Figure 7:**
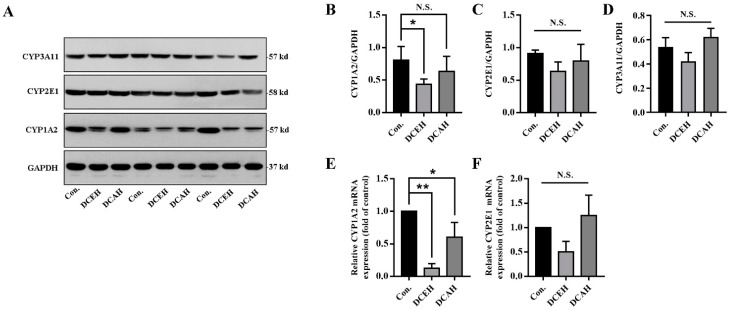
Protein level analysis of CYP1A2, CYP2E1 and CYP3A11 in the liver tissue. (**A**) Western blot results; (**B**–**D**) Western blot gray statistical analysis; (**E**,**F**) relative mRNA expression measured using q-PCR. (N.S., no significance, * *p* < 0.05 and ** *p* < 0.01, compared with control); Con.: control.

To further verify the predicted drug targets of DC, we compared the protein expression of CYP1A2, CYP2E1 and CYP3A11 in the liver tissue among normal, DCEH and DCAH samples. The results of Western blot revealed that DCEH significantly downregulated the transcription and expression of CYP1A2 and had no significant effect on CYP2E1 and CYP3A11, but tended to decrease when DCAH did not have a significant affect on these enzymes (Figure 7A–D). As shown in Figure 7E,F, DCEH and DCAH significantly downregulated the transcription of CYP1A2 but had no significant effect on CYP2E1.

## 3. Discussion

HILI is an impediment to the application of traditional Chinese medicine and has attracted the world’s attention. For further research and application, the first problem to be solved is the elucidation of the targets and mechanism of hepatotoxicity. According to previous reports, DC can induce liver injury, but the mechanism is unclear. Relying on network analysis, network toxicology can be used to predict the toxic components of traditional Chinese medicine (TCM) and explore the toxic mechanism, providing a new perspective and new ideas for toxicological research on TCM. Zhang et al. used serum pharmacochemistry and network toxicology to screen the potentially toxic components of *Radix Aconiti Lateralis* and explore the possible mechanism. It was found that 22 potential toxic components can affect Th17 cell differentiation and the Jak-STAT signaling pathway by regulating AKT1, IL2, F2, GSR and EGFR, which induce oxidative stress, metabolic disorders and cell apoptosis, eventually inducing liver damage in rats [20]. Jiang et al. constructed a hepatotoxicity interaction network of *Polygonum multiflorum* Thunb and used molecular docking to confirm the high binding activity of eight key toxic ingredients with 10 core targets, including mTOR, PIK3CA, AKT1 and EGFR, providing a theoretical foundation for the toxicity mechanisms of *Polygonum multiflorum* and its safe clinical application [21].

In this study, we first constructed the ‘toxicity-toxic components-toxic targets-toxic pathways’ network and researched the potential hepatotoxic mechanism of DC by collecting targets, pathway analysis and molecular docking. The network of ‘toxicity-toxic components-toxic targets-toxic pathways’ revealed that DC produced hepatotoxicity by participating in heterologous metabolism, REDOX metabolism, steroid metabolism, drug metabolism, heterocyclic metabolism, the synthesis of steroid hormones, cytochrome P450 metabolism, chemical carcinogenesis and bile secretion. Although there were many overlapping targets between compounds-targets and hepatotoxicity targets, the key targets were CYP1A2 and GSR according to the results of molecular docking. The results of the experiments on these cells indicated that alkaloids induced cytotoxicity more strongly than limonin. In vivo, the results of the staining and biochemical analysis indicated that DC can lead to hepatocyte injury and oxidation stress.

CYP1A2 is one of the most important CYP enzymes in the liver, accounting for 13% to 15% of hepatic CYP enzymes, and is responsible for the metabolism of polycyclic aromatic hydrocarbon and many clinical drugs [22]. If the activity of CYP1A2 is induced or inhibited, the normal physiological activity of hepatocytes would be affected, leading to hepatotoxicity [23,24]. Our results indicated that metabolites of *Dictamnus dasycarpus* can inhibit the activity of CYP1A2, even more strongly than α-naphthoflavone according to docking. In addition, CYP1A2 can protect against reactive oxygen production in the liver [25]. In our research, docking indicated that CYP1A2 activity was inhibited by alkaloids; WB and PCR analysis revealed that the expression of CYP1A2 was decreased, which can generate reactive oxygen production and accumulation, consistent with our animal experiment.

Glutathione reductase (GSR) is a key enzyme in the GSH redox cycle, reducing oxidized glutathione (GSSG) to reduce GSH [26], which can effectively eliminate reactive oxygen species (ROS), maintain intracellular redox balance and prevent oxidative damage to liver cells [27]. Several studies have shown that the furan ring can be metabolized by CYP3A4 to generate active intermediates that can combine with GSH, resulting in GSH depletion and hepatotoxicity [28,29,30]. As the main components of *Dictamnus dasycarpus*, furoquinoline alkaloids can be metabolized to dihydrofuroquinoline alkaloids by CYP3A4, consuming GSH simultaneously [31,32].

Our research first constructed the network of ‘toxicity-toxic components-toxic targets-toxic pathways’ of DC, providing further evidence for its hepatotoxicity. In our study, we found that GSR and CYP1A2 were key targets of hepatotoxicity induced by dihydrofuroquinoline alkaloids using network pharmacology. For verifying these results, we proved the cytotoxicity of the furoquinoline alkaloids in vitro. Furthermore, in vivo, we confirmed that DC can decrease the activity of GSH-Px, resulting in oxidative stress injury and inhibiting the expression of CYP1A2, which can induce hepatotoxicity. In other words, we drew a conclusion that the main toxic substances in DC were furoquinoline alkaloids, whose targets were CYP1A2 and GSR.

However, there are several questions worth exploring. The cause of the difference in toxicity between the aqueous extract and ethanol extract remains obscure. The compounds were so minor that we could not conduct further research on their deeper mechanism. Further investigation should be carried out on these compounds to determine whether we can obtain sufficient amounts via chemical biosynthesis. In addition to docking, it is more rigorous and reliable to verify the binding abilities of compounds and targets in vitro using molecular biological approaches, such as surface plasmon resonance (SPR) and biofilm interference (BLI) technology. It would be interesting and meaningful to explain the mechanism of CYP1A2-induced hepatotoxicity.

## 4. Materials and Methods

### 4.1. Materials

The root bark of *Dictamnus dasycarpus* Turcz. (Rutaceae) was supplied by the Anguo medicine market, Hebei province, China, and further identified by Professor Peng-Fei Tu, School of Pharmaceutical Sciences, Peking University, Beijing, China. A specimen has been deposited at the Herbarium of the Peking University Modern Research Center for Traditional Chinese Medicine (No. 201508150324).

### 4.2. Preparation of DC Extract

DC was pulverized into fine powders (50 mesh) using a medicinal material grinder and then extracted three times with 95% ethanol-water (*v*/*v*) and water by using the reflux extraction method for 1 h each. The extraction solutions were combined, filtered, evaporated and freeze-dried to obtain aqueous extract (DCA) and ethanol extract (DCE). The extraction efficiencies of DCA and DCE were 25% and 3.75%, respectively. DCA and DCE were suspended in 0.5% CMC-Na.

### 4.3. Network Pharmacology Analysis and Computer Docking Simulation

#### 4.3.1. Targets Collecting

On the basis of the results of in vivo metabolite identification in rats dosed with DC [10], 36 exposure constituents were selected for target prediction, the structures and molecules of which are listed in Table S1. The drug targets of the compound were found and selected from the database of TCMSP (https://tcmspw.com/tcmsp.php (accessed on 11 March 2021)), PharmMapper (http://lilab-ecust.cn/pharmmapper/index.html(accessed on 11 March 2021)), SwissTargetPrediction (http://www.swisstargetprediction.ch/(accessed on 11 March 2021)) and SEA (https://sea.bkslab.org/(accessed on 11 March 2021)). “Liver Injury” and “hepatotoxicity” were used as keywords to search for hepatotoxicity targets in CTD (http://ctdbase.org/(accessed on 11 March 2021)), and “marker/mechanism” was used as a limited property.

#### 4.3.2. Establishment of Protein–Protein Interaction

The hepatotoxicity targets of the candidate compounds were obtained by comparing the targets of the candidate compounds and hepatotoxicity targets. The hepatotoxicity targets of the candidate compounds were imported into the STRING database, the species was selected as “Homo sapiens”, and the scoring condition was set as >0.40. Discrete targets were removed, and the protein interaction network was obtained and saved as TSV format files. The TSV format file was imported into Cytoscape 3.7.2 software to conduct a visual analysis of the network. The hepatotoxicity targets of the candidate compounds were imported into the David online analysis platform for GO and KEGG enrichment analyses, and the results of GO and KEGG enrichment analyses were selected and sorted under the condition of *p* < 0.05.

The data of “DC-candidate compounds”, “candidate compounds-targets”, “targets-pathways”, and “pathways-hepatotoxicity” were collected in Excel and imported into Cytoscape 3.7.2 software to construct the network of “potential toxic components-toxic targets-toxic pathways” of DC-hepatotoxicity. The network characteristics were analyzed using “Network analysis”.

#### 4.3.3. Docking

Schrodinger 11.8 software was used to construct the molecular docking model of the key targets for molecular docking with the components of DC exposed in vivo. First, the protein structures of the key targets of DC hepatotoxicity were downloaded from the PDB database, and complex crystal structures with small ligand molecules and resolutions of less than 3 Å were selected. At the same time, the related best ligand structure was downloaded as the positive control. Schrodinger 11.8 software was used to conduct dehydration, hydrogenation, residue completion and pocket generation of the protein. The structure of the candidate compounds was optimized, and then the docking experiment was carried out.

### 4.4. Cell Experiments

#### 4.4.1. Cell Culture

The HepG2 cell line was purchased from the Cell Bank of Peking Union Medical College in China. The cells were cultured in Dulbecco’s modified Eagle’s medium containing 10% fetal bovine serum, 100 U/mL penicillin and 100 mg/mL streptomycin. The cell culture conditions were maintained at 37 °C in a 5% CO_2_ incubator under 95% absolute humidity.

#### 4.4.2. MTT Analysis

Cell viability was assessed using a 3-(4,5-dimethyl thiazol-2-yl)-2,5-diphenyl tetrazolium bromide (MTT) solution (Solarbio, Beijing, China). Adriamycin was selected as the positive control and 1% DMSO was the negative control. The compounds were prepared in a stock solution of 20 mM. HepG2 cells were treated for 48 h, the culture supernatant was removed, and a staining solution containing MTT (5 mg/mL) was added to each well. The cells were then incubated for 4–6 h in the dark at 37 °C. Thereafter, dimethyl sulfoxide (DMSO) was used to dissolve formazan crystals, and the absorbance at 570 nm was determined spectrophotometrically on an ELX800 UV universal microplate reader (Bio-Tek, Winooski, VT, USA). Data were graphically displayed using GraphPad Prism (version 6.0).

### 4.5. Animal Experiments

#### 4.5.1. Animals

Male and female ICR mice (6–7 weeks of age, 18–22 g) were obtained from the Laboratory Animal Center of the Peking University Health Science Center (Beijing, China). The mice were housed at 25 °C and 60 ± 5% humidity, with a 12 h dark–light cycle. They were fed standard laboratory food and water ad libitum. All mice were fasted for 12 h but had free access to water prior to the sacrifice. The animal facilities and protocols were approved by the Animal Care and Use Committee of the Peking University Health Science Center (LA2015061). All procedures were performed in accordance with the Guide for the Care and Use of Laboratory Animals (National Institute of Health).

#### 4.5.2. Mice and Drug Administration for the Toxicity Test

After acclimatization for one week, 70 male mice and 70 female mice were randomly divided into seven groups (*n* = 10): normal group, DCE high-dose group (DCEH), DCE middle-dose group (DCEM), DCE low-dose group (DCEL), DCA high-dose group (DCAH), DCA middle-dose group (DCAM) and DCA low-dose group (DCAL). DCEL, DCEM and DCEH were orally dosed with 235 mg/kg/d, 470 mg/kg/d and 940 mg/kg/d DCE for two weeks, respectively. DCAL, DCAM, and DCAH were orally dosed with 1.56 g/kg/d, 3.12 g/kg/d and 6.24 g/kg/d of DCA for two weeks, respectively. All the mice were weighed and recorded every 3 to 4 days.

#### 4.5.3. Sample Collection

All blood samples were collected in a heparinized tube by extirpating the eyeball, and subsequently, all animals were sacrificed immediately by cervical vertebral dislocation. The obtained blood samples were centrifuged at 5000 rpm for 10 min at 4 °C. Then, the serum supernatants were collected and stored at −80 °C for further analysis.

After collecting the blood, an intact liver was obtained, cleaned with normal saline and weighed. Three mice per group were fixed in 4% paraformaldehyde for the tissue section experiment, and the others were immediately frozen in liquid nitrogen and then stored at −80 °C.

#### 4.5.4. Histology Assay

The livers were embedded in paraffin and fixed with 4% paraformaldehyde. The paraffin sections (5 μm) of liver tissues were stained with routine hematoxylin/eosin (H&E) staining. All histological images were obtained using an Eclipse Ti-SR fluorescence microscope (Nikon, Japan) and Pannoramic MIDI (3DHISTECH Ltd., Budapest, Hungary) and analyzed using Image J software version 1.52a (National Institutes of Health, Bethesda, MD, USA).

#### 4.5.5. Biochemical Analysis

Frozen liver tissue (0.1 g) was added to 1 mL of normal saline, and a 10% tissue homogenate was obtained using a homogenizer (FastPrep-24™ 5G, MP, Santa Ana, CA, USA). The homogenate was centrifuged at 12,000 rpm at 4 °C for 15 min, and the supernatant was taken for protein quantification and detection of the activities of superoxide dismutase (SOD), glutathione peroxidase (GSH-Px) and the levels of malondialdehyde (MDA), which were examined with detection kits following the Jiancheng Bioengineering Institute’s protocol (Nanjing, China).

The activity of alanine aminotransferase (ALT) and aspartate aminotransferase (AST) in serum was determined using an ELISA kit according to the manufacturer’s instructions (Sigma-Aldrich, Ltd., St. Louis, MO, USA). The concentrations of total protein (TP), albumin (ALB), alkaline phosphatase (ALP), total bilirubin (T-Bil) and direct bilirubin (D-Bil) in the serum samples were measured following the Jiancheng Bioengineering Institute’s protocol (Nanjing, China).

#### 4.5.6. Western Blot and Real-Time q-PCR Analysis

The 10% tissue homogenate of livers was added to ice-cold RIPA buffer containing 1 mM PMSF (Macgene, Beijing, China) to obtain the tissue lysate. Whole proteins were collected by centrifugation at 13,000 rpm at 4 °C for 20 min. The concentration of the total proteins was detected using a protein assay kit (TransGen, Beijing, China). Equal concentrations of protein solutions were separated by 10% SDS-polyacrylamide gel electrophoresis and transferred to PVDF membranes. Next, the PVDF membranes were blocked with 5% skim milk at 25 °C for 60 min. Subsequently, the membranes were incubated with primary antibodies against GAPDH (CST, Boston, MA, USA), CYP1A2 (CST, USA), CYP2E1 (CST, USA) and CYP3A11 (anti-CYP3A11, CST, USA) at 4 °C for 12 h. After incubation with a second antibody at room temperature for 1 h, the membranes were developed using SuperSignal West Femto Maximum Sensitivity Substrate. The protein bands were imaged using a Tanon 5200 Imaging Analysis System (Tanon, Shanghai, China). The ImageJ software was used to calculate the relative gray of bands.

The liver tissues were processed for the isolation of total RNA by using TRIzol reagent (Invitrogen, Carlsbad, CA, USA) according to the manufacturer’s protocol. The RNA concentration was determined, and the quality of the isolated RNA was assessed using the 260/280 nm absorbance ratio (1.8–2.0 indicates a highly pure sample). The RNA pellet was stored at −80 °C until further use. According to the instructions of the supplier, we carried out inverse transcription utilizing MMLV reverse transcriptase (Takara Biotechnology, Dalian, China). The resulting reverse transcription products were stored at −80 °C until the assay. The polymerase chain reaction was carried out using an initial denaturation at 95 °C for 5 min, followed by 40 cycles of 95 °C for 15 s and 65 °C for 30 s using a qPCR instrument of Agilent. The sequences of the forward and reverse primers are summarized in Table 2. Relative transcriptional levels normalized to those of β-actin were calculated using the comparative 2^−ΔΔCT^ method.

### 4.6. Statistical Analysis

All values were expressed as mean ± SD (standard deviation). Statistical analyses were performed using a one-way analysis of variance with GraphPad Prism 6.0 software. Multiple comparisons were performed by comparing the mean of each column with the mean of every other column. Student’s *t*-test was used without correction for multiple comparisons. A *p* value < 0.05 was considered to be significant.

## 5. Conclusions

In conclusion, we constructed a “potential toxic components-toxic target-toxic pathway” using a network toxicology method and verified the potential mechanism of the hepatotoxicity of *Dictamnus dasycarpus* by molecular docking and animal experiments. Our results showed that the targets of DC included CYP1A2 and GSR, participating in heterologous steroid metabolism, REDOX metabolism, drug metabolism, heterocyclic metabolic process, the synthesis of steroid hormone, cytochrome P450 metabolism, chemical carcinogens and bile secretion pathways. Dictamni Cortex can cause oxidative stress and a decrease in CYP1A2, inducing liver injury. These results further explain the mechanism of hepatotoxicity induced by *Dictamnus dasycarpus*, providing a basic theory to explore and prevent hepatotoxicity in the clinical usage of *Dictamnus dasycarpus*.

## Figures and Tables

**Table 1:** The docking scores of targets with candidate compounds.

Compound ID	Structure	FXR	TNF	PXR	ALB	CDK2	IDH2	GSR	P53	CYP3A4	CYP1A2	CYP2C9	CYP2E1
**1**	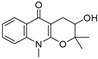	−8.046	−5.288	−8.328	−7.729	−6.825	−9.542	**−4.485**	−7.169	−6.608	−7.161	−4.703	—
**2**	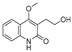	−6.727	−5.762	−7.918	−8.4	−7.972	−9.713	−3.551	−7.924	−5.827	−7.163	−4.029	—
**3**	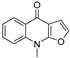	−7.409	−4.142	−7.445	−7.908	−5.996	−7.734	−3.854	−6.462	−1.06	−6.816	−4.68	—
**4**	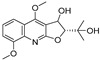	−9.303	−6.333	−8.841	−10.145	−7.922	−10.494	**−4.549**	−8.136	−6.948	**−9.582**	−5.299	—
**5**	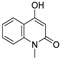	−6.047	−3.995	−7.58	−6.798	−5.826	−5.878	−2.933	−6.069	−4.201	−6.071	−4.637	−5.08
**6**	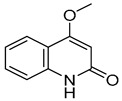	−5.888	−4.087	−7.094	−5.954	−6.581	−8.858	−2.504	−7.434	−4.508	−6.489	−3.953	−5.053
**7**	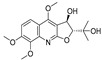	−7.845	−6.365	−8.852	−8.813	−7.474	−9.584	**−4.281**	−6.978	−7.041	**−9.526**	−6.167	—
**8**	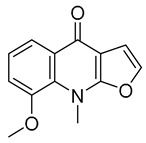	−6.641	−4.495	−7.13	−6.695	−7.396	−5.76	−3.461	−5.554	−0.951	−6.982	−3.89	—
**9**	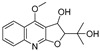	−9.512	−6.213	**−9.92**	−9.254	−7.648	−10.26	**−5.467**	−8.249	−7.687	**−9.073**	−5.834	—
**10**	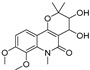	−9.821	−6.217	−9.357	−9.505	−6.283	−10.52	**−4.948**	−8.345	−6.562	**−9.218**	−5.132	—
**11**	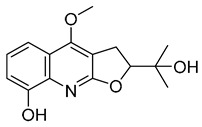	−8.83	−5.672	−9.025	−9.052	−7.662	−9.652	**−4.207**	−8.042	−6.212	−8.858	−5.403	—
**12**	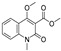	−7.32	−4.448	−7.041	−8.101	−5.494	−8.236	—	−5.944	−4.084	−7.276	−4.318	—
**14**	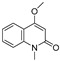	−6.635	−4.315	−6.949	−6.927	−6.177	−5.952	−3.215	−6.383	−3.126	−6.683	−4.446	—
**13**	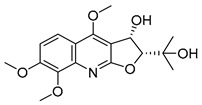	−8.042	−5.915	−9.189	−7.942	−7.040	−10.471	**−4.931**	−7.477	−6.799	**−9.261**	−6.167	—
**15**	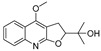	−8.853	−5.738	−8.561	−9.658	−6.392	−9.573	**−4.075**	−7.54	−6.542	−8.801	−3.841	—
**16**	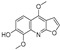	−7.221	−4.811	−7.186	−7.859	−8.648	−8.578	−3.901	−7.09	−5.673	−7.112	—	—
**17**	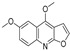	−7.041	−4.449	−6.692	−7.241	−7.073	−5.56	−3.884	−6.106	−5.164	−7.29	—	—
**18**	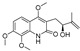	−8.798	−6.439	−8.404	−8.157	−8.706	−8.803	−3.133	−6.597	−6.532	−7.991	—	—
**19**	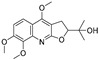	−7.439	−5.675	−8.039	−8.574	−6.163	−9.865	**−4.202**	−7.072	−5.907	**−9.236**	—	—
**20**	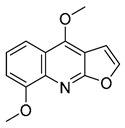	−6.405	−4.389	−6.581	−7.598	−5.329	−6.919	−3.303	−6.737	−4.824	−6.146	—	—
**21**	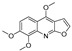	−5.114	−4.593	−5.714	−5.292	−4.549	−6.365	−3.32	−5.619	−4.22	−7.011	—	—
**22**	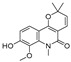	−8.882	−5.915	−8.8	−8.998	−9.098	−8.728	−3.594	−7.598	−6.348	−8.297	—	—
**23**	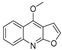	−6.749	−4.382	−6.552	−10.834	−5.792	−6.789	−3.916	−9.861	−4.767	−7.665	—	—
**24**	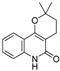	−8.314	−6.522	−7.407	−7.875	−8.172	−10.103	−3.942	−8.651	−5.46	−7.555	—	—
**25**	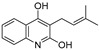	−6.658	−6.363	−7.61	−6.733	−7.297	−7.867	−2.334	−7.687	−5.699	−7.169	—	—
**26**	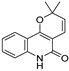	−7.887	−6.064	−7.79	−7.863	−6.917	−10.625	−3.979	−8.332	−5.645	−6.882	—	—
**27**	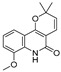	−8.3	−5.228	−8.438	−6.799	−8.524	−8.313	−3.379	−7.344	−5.454	−7.437	—	—
**28**	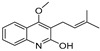	−8.122	−5.349	−7.91	−6.456	−8.923	−10.824	−4.135	−8.317	−6.669	−8.258	—	—
**29**	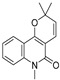	−8.156	−5.049	−7.571	−7.455	−5.983	−8.465	−3.472	−7.264	−5.663	−7.515	—	—
**30**	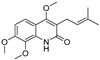	−8.774	−5.239	−7.954	−7.224	−9.76	−10.38	−3.901	−5.585	−5.31	−7.276	—	—
**31**	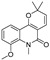	−8.443	−4.814	−6.797	−7.803	−7.696	−8.308	−3.278	−7.189	−5.506	−8.345	—	—
**32**	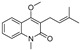	−8.092	−4.395	−7.071	−7.457	−5.573	−8.168	−3.439	−6.715	−6.408	−7.98	−4.367	—
**33**	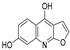	−7.411	−4.922	−6.544	−7.948	−9.275	−8.186	−3.748	−6.302	−5.755	−7.006	—	—
**34**	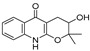	−10.404	−5.62	−8.012	−7.523	−7.125	−9.707	**−4.316**	−8.043	−6.531	−7.345	—	—
**35**	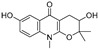	−7.204	−5.489	−7.801	−6.961	−6.152	−8.646	**−4.529**	−5.604	−6.075	−7.993	—	—
**36**	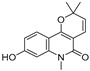	−8.285	−5.693	−7.836	−7.065	−8.184	−7.151	−3.512	−7.228	−5.787	−7.471	—	—
Obacunone	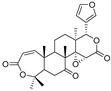	—	−4.734	−9.098	—	−2.579	—	−1.778	−3.543	−3.828	—	—	—
Fraxinellone	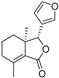	−8.418	−4.264	−9.335	−7.529	−5.352	−8.29	−3.661	−6.916	−0.906	−6.557	−3.226	—

Note: the “—” indicates that the compound cannot dock with the target; **the bold** values are higher than the optimal ligand docking scores.

**Table 2:** The sequences of the forward and reverse primers of the target gene.

Gene Name	Sequences
*Actb*	Forward: CCTAGCACCATGAAGATCAAGAT
	Reverse: ACTCATCGTACTCCTGCTTGCT
*Cyp2e1*	Forward: TCCAGAGACATTTAAACCTGAGC
	Reverse: GACAAAAGCAGAAACAGTTCCAT
*Cyp1a2*	Forward: GCTTCTCCATAGCCTCGGAC
	Reverse: CTGGCTGACTGGTTCGAAGT

## Data Availability

All data are contained in the article and the Supplementary Materials.

## Supplementary Materials

The following supporting information can be downloaded at: https://www.mdpi.com/article/10.3390/molecules28135045/s1, Table S1: Candidate compounds and the degree values; Table S2: The targets of candidate compounds related with hepatotoxicity; Table S3: GO analysis results; Table S4: KEGG analysis results; Table S5: The crystal structure of target protein and the docking fraction with the optimal ligand; Figure S1: The “candidate compounds-targets” network; Figure S2: The body weight change and relative liver weight of female mice; Figure S3: The H&E stain (A) and serum biochemical indexes (B) of female mice.

## Sample Availability

Samples of the compounds and extracts are available from the authors.
