# Supplementary material for: Exploring the Mechanism of Hepatotoxicity Induced by Dictamnus dasycarpus Based on Network Pharmacology, Molecular Docking and Experimental Pharmacology
Source: Molecules. 2023 Jun 28;28(13):5045. doi: 10.3390/molecules28135045 (PMC10343517; doi:10.3390/molecules28135045)
Supplement: Supplementary file 1 [file molecules-28-05045-s001.zip › molecules-2442037-SI.pdf]

Table S1 Candidate compounds and the degree values

Table S2 The targets of candidate compounds related with hepatotoxicity

Table S3 GO analysis results

Table S4 KEGG analysis results

Table S5 The crystal structure of target protein and the docking fraction with the optimal ligand

Figure S1 The "candidate compounds-targets" network

Figure S2 The body weight change and relative liver weight of female mice

Figure S3 The H&E stain **(A)** and serum biochemical indexes **(B)** of female mice

Table S1 Candidate compounds and the degree values

ID	Name	Degree
1	Ribalinine	153
2	3-(2-hydroxyethyl)-4-methoxy-2(1*H*)-quinolinone	111
3	Isodictamnine	29
4	8-methoxymyrtopsine	188
5	4-hydroxy-1-methyl-2(1*H*)-quinolinone	43
6	4-methoxy-2(1*H*)-quinolinone	264
7	(+)-cis-7,8-dimethoxymyrtopsine	12
8	*Iso*-*γ*-fagarine	79
9	Myrtopsine	105
10	Araliopsinine	151
11	8-hydroxy-platydesmine	93
12	1,2-dihydro-4-methoxy-1-methyl-2-oxo-, methyl ester-3-quinolinecarboxylic acids	98
13	(+)-7,8-dimethoxymyrtopsine	128
14	4-methoxy-1-methyl-2(1*H*)-quinolinone	70
15	Platydesmine	104
16	Haplopine	80
17	6-methoxydictamnine	43
18	Dasycarine D	139
19	7,8-dimethoxyplatydesmine	108
20	*γ*-fagarine	47
21	Skimmianine	62
22	2,6-dihydro-8-hydroxy-7-methoxy-2,2,6-trimethyl-5*H*-pyrano[3,2-c]quinolin-5-one	108
23	Dictamnine	34
24	Dihydroflindersine	104
25	2,4-hydroxy-3-(3'-methyl-2'-butenyl)-quinolin	140
26	Flindersine	100
27	8-methoxyflindersine	90
28	2-hydroxy-4-methoxy-3-(3'-methyl-2'-butenyl)-quinolin	147

29	2,6-dihydro-2,2,7-trimethyl-5*H*-pyrano[3,2-c]quinolin-5 one	73
30	Preskimmianine	168
31	8-methoxy-*N*-methylflindersine	71
32	*N*-metilatanina	24
33	Furo[2,3-b]quinoline-4,7-diol	61
34	2,3,4,10-tetrahydro-3-hydroxy-2,2-dimethyl-5*H*-pyrano[2,3-b]quinolin-5-one	137
35	2,3,4,10-tetrahydro-3,7-dihydroxy-2,2,10-trimethyl-5*H*-pyrano[2,3-b]quinolin-5-one	135
36	2,6-dihydro-8-hydroxy-2,2,6-trimethyl-5*H*-Pyrano[3,2-c]quinolin-5-one	90

Table S2 The targets of candidate compounds related with hepatotoxicity

ID	Uniprot ID	Gene name	Protein name
1	P02766	TTR	Transthyretin
2	P06737	PYGL	Glycogen phosphorylase, liver form
3	P00439	PAH	Phenylalanine-4-hydroxylase
4	P00374	DHFR	Dihydrofolate reductase
5	P11712	CYP2C9	Cytochrome P450 2C9
6	P05089	ARG1	Arginase-1
7	P02768	ALB	Serum albumin
8	Q00796	SORD	Sorbitol dehydrogenase
9	P04179	SOD2	Superoxide dismutase [Mn], mitochondrial
10	P00491	PNP	Purine nucleoside phosphorylase
11	P09211	GSTP1	Glutathione S-transferase P
12	P80188	LCN2	Neutrophil gelatinase-associated lipocalin
13	P00390	GSR	Glutathione reductase, mitochondrial
14	Q6ZQW0	IDO2	Indoleamine 2,3-dioxygenase 2
15	Q71U36	TUBA1A	Tubulin alpha-1A chain
16	P36897	TGFBR1	TGF-beta receptor type-1
17	P07451	CA3	Carbonic anhydrase 3
18	O00204	SULT2B1	Sulfotransferase 2B1

19	P49888	SULT1E1	Sulfotransferase 1E1
20	Q14994	NR1I3	Nuclear receptor subfamily 1 group I member 3
21	Q96RI1	NR1H4	Bile acid receptor
22	P08253	MMP2	72 kDa type IV collagenase
23	P32298	GRK4	G protein-coupled receptor kinase 4
24	P02774	GC	Vitamin D-binding protein
25	P01375	TNF	Tumor necrosis factor
26	O60427	FADS1	Acyl-CoA
27	P05177	CYP1A2	Cytochrome P450 1A2
28	P35869	AHR	Aryl hydrocarbon receptor
29	P08183	ABCB1	ATP-dependent translocase ABCB1
30	Q92887	ABCC2	Canalicular multispecific organic anion transporter 1
31	O60656	UGT1A9	UDP-glucuronosyltransferase 1A9
32	P08684	CYP3A4	Cytochrome P450 3A4
33	P05181	CYP2E1	Cytochrome P450 2E1
34	P17931	LGALS3	Galectin-3
35	P20309	CHRM3	Muscarinic acetylcholine receptor M3

Table S3 GO analysis results

**Type**	**No.**	**Name**	**Target number**	**-log$_{10}$*P***
BP	GO:0006805	xenobiotic metabolic process	7	8.00
	GO:0016098	monoterpenoid metabolic process	4	6.82
	GO:0055114	oxidation-reduction process	11	6.78
	GO:0008202	steroid metabolic process	5	5.79
	GO:0017144	drug metabolic process	4	4.67
	GO:0042493	response to drug	7	4.53
	GO:0042737	drug catabolic process	3	4.23
	GO:0046483	heterocycle metabolic process	3	4.23
	GO:0071222	cellular response to lipopolysaccharide	5	4.11

	GO:0070989	oxidative demethylation	3	3.59
	GO:0042738	exogenous drug catabolic process	3	3.59
	GO:0019373	epoxygenase P450 pathway	3	3.22
	GO:0046686	response to cadmium ion	3	2.94
	GO:2001237	negative regulation of extrinsic apoptotic signaling pathway	3	2.57
	GO:0000302	response to reactive oxygen species	3	2.55
MF	GO:0008144	drug binding	7	8.08
	GO:0019825	oxygen binding	6	7.45
	GO:0016491	oxidoreductase activity	7	5.58
	GO:0004497	monooxygenase activity	5	5.27
	GO:0005506	iron ion binding	6	4.89
	GO:0034875	caffeine oxidase activity	3	4.63
	GO:0020037	heme binding	5	3.80
	GO:0016712	oxidoreductase activity, acting on paired donors, with incorporation or reduction of molecular oxygen, reduced flavin or flavoprotein as one donor, and incorporation of one atom of oxygen	3	3.39
	GO:0008395	steroid hydroxylase activity	3	2.91
	GO:0004879	RNA polymerase II transcription factor activity	3	2.62
	GO:0002060	purine nucleobase binding	2	2.40
	GO:0016705	oxidoreductase activity, acting on paired donors, with incorporation or reduction of molecular oxygen	3	2.23
	GO:0050294	steroid sulfotransferase activity	2	2.10
	GO:0004887	thyroid hormone receptor activity	2	1.80
	GO:0032052	bile acid binding	2	1.80
CC	GO:0070062	extracellular exosome	17	4.87
	GO:0005829	cytosol	17	3.96
	GO:0005615	extracellular space	10	3.21
	GO:0031090	organelle membrane	3	1.94
	GO:0043231	intracellular membrane-bounded organelle	5	1.71

	GO:0005789	endoplasmic reticulum membrane	6	1.68
	GO:0043209	myelin sheath	3	1.49
	GO:0070062	extracellular exosome	17	4.87

Table S4 KEGG analysis results

No.	Pathway	Targets number	-log$_{10}$*P*	Target name
hsa00140	Steroid hormone biosynthesis	6	5.60	SULT2B1, SULT1E1, CYP1A2, CYP2E1, UGT1A9, CYP3A4
hsa00982	Drug metabolism - cytochrome P450	6	5.26	CYP2C9, GSTP1, CYP1A2, CYP2E1, UGT1A9, CYP3A4
hsa00980	Metabolism of xenobiotics by cytochrome P450	6	5.07	CYP2C9, GSTP1, CYP1A2, CYP2E1, UGT1A9, CYP3A4
hsa05204	Chemical carcinogenesis	6	4.91	CYP2C9, GSTP1, CYP1A2, CYP2E1, UGT1A9, CYP3A4
hsa00591	Linoleic acid metabolism	4	3.73	CYP2C9, CYP1A2, CYP2E1, CYP3A4
hsa00830	Retinol metabolism	4	2.72	CYP2C9, CYP1A2, UGT1A9, CYP3A4
hsa01100	Metabolic pathways	12	2.38	DHFR, CYP2C9, PNP, ARG1, PAH, CYP1A2, SORD, CYP2E1, PYGL, UGT1A9, CYP3A4, IDO2
hsa04976	Bile secretion	3	1.53	ABCB1, ABCC2, NR1H4

Table S5 The crystal structure of target protein and the docking fraction with the optimal ligand

Target	PDB	Ligand	Docking score
FXR	1OSV	Obeticholic acid	-14.688
TNF	2AZ5	6,7-dimethyl-3-[[methyl[2-[methyl[[1-	-7.222

		[3-(trifluoromethyl)phenyl]-1*H*-indol-3-yl]methyl]amino]ethyl]amino]methyl]-4*H*-1-benzopyran-4-one	
PXR	3R8D	6-chloro-2-(1-furo[2,3-c]pyridin-5-yl-ethylsulfanyl)-pyrimidin-4-ylamine	-9.451
ALB	6HSC	Aristolochic acid	-12.171
CDK2	6INL	Cvt-313	-11.519
IDH2	5I96	Enasidenib	-15.831
GSR	1XAN	3,6-dihydroxy-xanthene-9-propionic acid	-3.979
P53	4XZV	Maltose	-11.183
CYP1A2	2HI4	Alpha-naphthoflavone	-8.948
CYP2C9	4NZ2	(2*R*)-*N*-[4-(3-bromophenyl)sulfonyl-2-chlorophenyl]-3,3,3-trifluoro-2-hydroxy-2-methylpropanamide	-6.308
CYP3A4	2V0M	Ketoconazole	-8.386
CYP2E1	3E6I	1*H*-indazole	-5.681

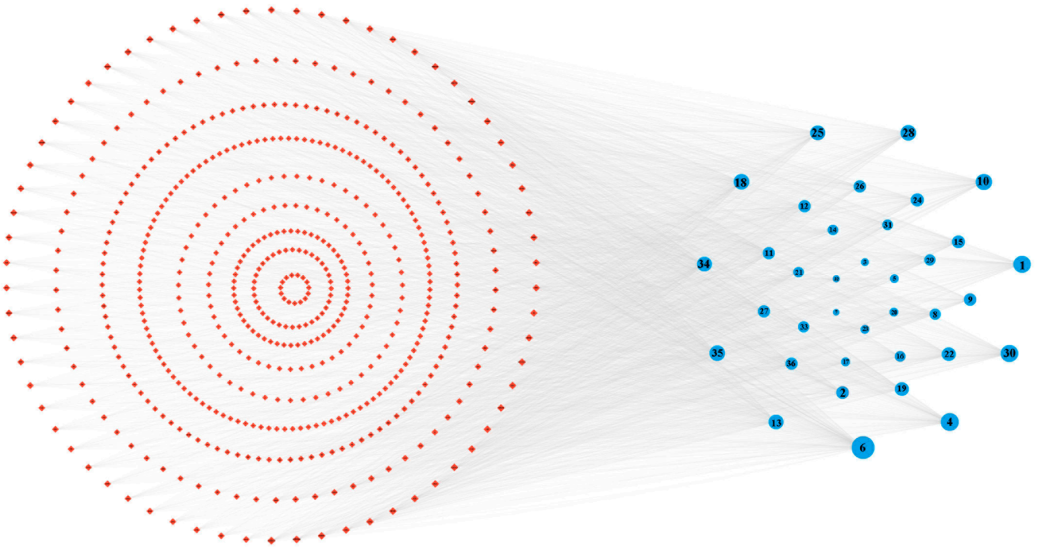

Figure S1 The “candidate compounds-targets” network

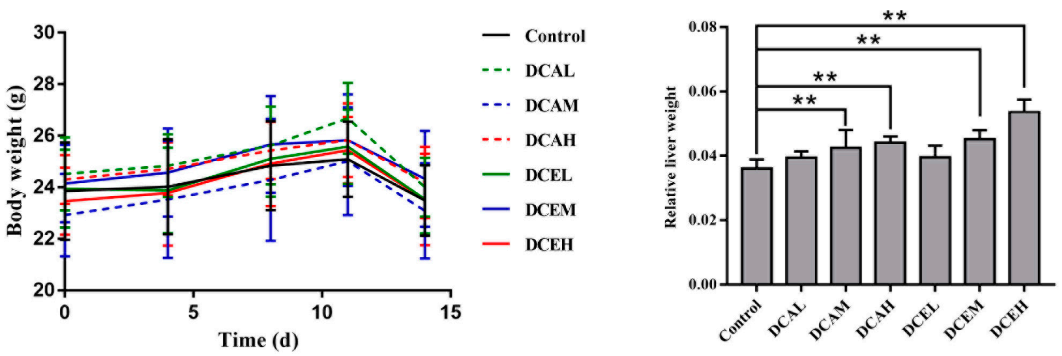

Figure S2 The body weight change and relative liver weight of female mice

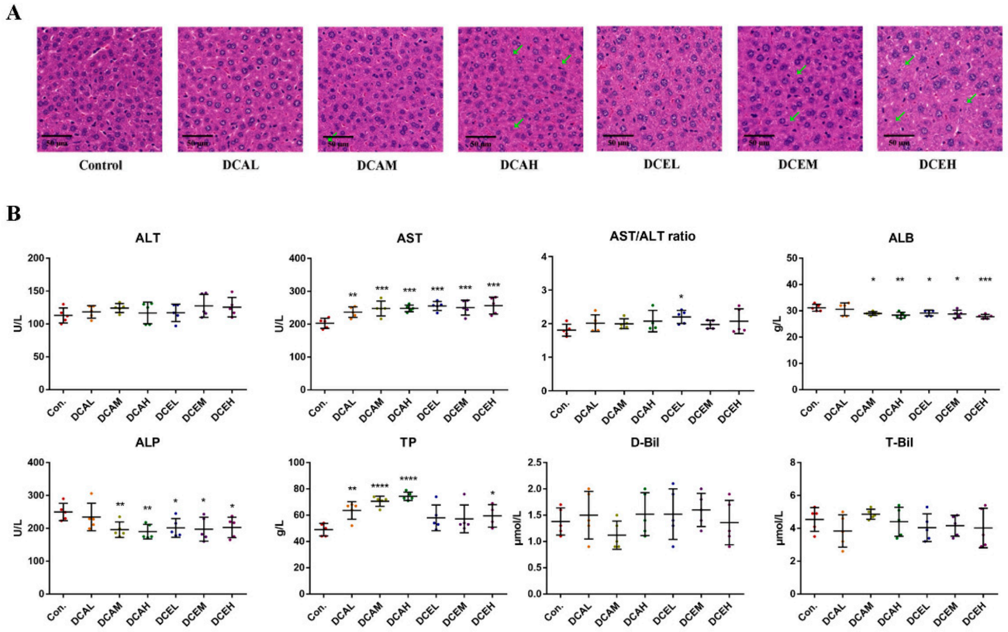

Figure S3 The H&E stain and weight change and relative liver weight of female mice
